# Differentiation of COVID-19 from other types of viral pneumonia and severity scoring on baseline chest radiographs: Comparison of deep learning with multi-reader evaluation

**DOI:** 10.1371/journal.pone.0328061

**Published:** 2025-07-29

**Authors:** Nastaran Enshaei, Arash Mohammadi, Farnoosh Naderkhani, Nick Daneman, Rawan Abu Mughli, Reut Anconina, Ferco H. Berger, Robert Andrew Kozak, Samira Mubareka, Ana Maria Villanueva Campos, Keshav Narang, Thayalasuthan Vivekanandan, Adrienne Kit Chan, Philip Lam, Nisha Andany, Anastasia Oikonomou

**Affiliations:** 1 Concordia Institute for Information Systems Engineering, Concordia University, Montreal, Quebec, Canada; 2 Department of Medicine, Division of Infectious Diseases, Sunnybrook Health Sciences Centre, University of Toronto, Toronto, Canada; 3 Department of Medical Imaging, Sunnybrook Health Sciences Centre, University of Toronto, Toronto, Canada; 4 Biological Sciences Platform, Sunnybrook Research Institute and Shared Hospital Laboratory, Toronto, Canada; 5 Department of Microbiology, Sunnybrook Health Sciences Centre, University of Toronto, Toronto, Canada; Najran University College of Computer Science and Information Systems, SAUDI ARABIA

## Abstract

Chest X-ray (CXR) imaging plays a pivotal role in the diagnosis and prognosis of viral pneumonia. However, distinguishing COVID-19 CXRs from other viral infections remains challenging due to highly similar radiographic features. Most existing deep learning (DL) models focus on differentiating COVID-19 from community-acquired pneumonia (CAP) rather than other viral pneumonias and often overlook baseline CXRs, missing the critical window for early detection and intervention. Moreover, manual severity scoring of COVID-19 CXRs by radiologists is subjective and time-intensive, highlighting the need for automated systems. This study introduces a DL system for distinguishing COVID-19 from other viral pneumonias on baseline CXRs acquired within three days of PCR testing, and for automated severity scoring of COVID-19 CXRs. The system was developed using a dataset of 2,547 patients (808 COVID-19, 936 non-COVID viral pneumonia, and 803 normal cases) and validated externally on several publicly accessible datasets. Compared to four experienced radiologists, the model achieved higher diagnostic accuracy (76.4% vs. 71.8%) and enhanced COVID-19 identification (F1-score: 74.1% vs. 61.3%), with an AUC of 93% for distinguishing between viral pneumonia and normal cases, and 89.8% for differentiating COVID-19 from other viral pneumonias. The severity-scoring module exhibited a high Pearson correlation of 93% and a low mean absolute error (MAE) of 2.35 compared to the radiologists’ consensus. External validation on independent public datasets confirmed the model’s generalizability. Subgroup analyses stratified by patient age, sex, and severity levels further demonstrated consistent performance, supporting the system’s robustness across diverse clinical populations. These findings suggest that the proposed DL system could assist radiologists in the early diagnosis and severity assessment of COVID-19 from baseline CXRs, particularly in resource-limited settings.

## Introduction

Viral lung infections pose significant public health challenges due to their rapid transmission and potential to cause severe respiratory illness. The clinical severity can range from mild symptoms to life-threatening complications, particularly in high-risk groups such as the elderly, young children, and those with compromised immune systems. During the COVID-19 pandemic, healthcare systems worldwide were severely strained, facing overwhelming patient load and shortages in clinical resources [1]. Although widespread vaccination has helped transition COVID-19 into a global endemic phase [2], the continued circulation of SARS-CoV-2, along with the resurgence of other respiratory viruses [3] that were previously suppressed during the pandemic, highlights the need for prompt diagnosis and timely intervention to manage seasonal surges and mitigate their impact on healthcare systems. Accurate differentiation between COVID-19 and other viral respiratory infections is clinically important as targeted SARS-CoV-2-specific treatments such as antivirals and monoclonals can be administered to moderate or severe cases to reduce mortality [4,5].

While Polymerase Chain Reaction (PCR) remains the gold standard for COVID-19 diagnosis and rapid testing is widely accessible in many regions, chest imaging is recommended as a supplementary diagnostic tool in resource-limited settings or when symptoms worsen despite negative PCR results [6,7]. CXR stands out as a cost-effective, rapid, and widely available imaging modality with lower radiation exposure compared to CT. Portable CXR units can also be used in high-risk areas such as emergency and isolation rooms, minimizing transmission risks and enabling bedside monitoring of critically ill patients [8,9].

For these reasons, despite its lower sensitivity compared to CT [10], CXR remains the first-line imaging modality for COVID-19, aiding not only in the initial assessment but also in ruling out other non-infectious related entities or complications such as secondary bacterial pneumonia [8,11]. Moreover, CXR-based evaluation of lung involvement and infection patterns has proven to be a valuable prognostic indicator for disease severity and clinical outcomes [12,13]. However, the manual quantification of COVID-19 severity on CXRs is subjective and time-intensive for radiologists, driving interest in DL systems for automated severity scoring [14,15].

Several studies have explored DL models for COVID-19 diagnosis using CXRs, with their performance underperforming [16], outperforming [17,18], or being comparable [19,20] to those of radiologists. However, most of these models have relied on CXRs obtained during the advanced stages of illness, when radiographic findings are more pronounced, rather than utilizing baseline CXRs captured at initial presentation. This early time point is crucial for the timely differentiation of COVID-19 from other viral infections, even before PCR test results are available. Furthermore, these models mainly focus on distinguishing COVID-19 from CAP cases rather than non-COVID-19 viral pneumonia. This is a significant challenge since COVID-19 shares highly similar radiographic features with other viral pneumonias, making accurate diagnosis difficult even for expert thoracic radiologists. Studies focusing on distinguishing COVID-19 CXRs from other types of viral pneumonia [21,22] often neglect to provide the timing of image acquisition and disease stage, which are crucial for assessing the real-world applicability of CXR-based diagnostic models.

To address these gaps, this study introduces a DL-based framework for distinguishing COVID-19 from other viral pneumonias and normal cases using baseline CXRs. The framework also incorporates a COVID-19 severity scoring module, providing a unified approach for early diagnosis and severity assessment. The key contributions of this study are as follows:

- *Development of a DL framework for early diagnosis and severity scoring:* We propose an integrated DL system for distinguishing COVID-19 from non-COVID-19 viral pneumonia and normal cases using baseline CXRs, while also predicting severity scores for COVID-19 cases. Our study differs from previous research in two key aspects: (i) it uses baseline CXRs captured within three days of PCR testing, whereas most prior studies focus on images from advanced disease stages; and (ii) it tackles the more challenging task of differentiating COVID-19 from other viral pneumonias, including those caused by non-COVID-19 coronaviruses, while prior work has largely focused on distinguishing COVID-19 from CAP cases.
- *Comprehensive comparison with radiologists:* The diagnostic and severity scoring performance of the proposed system was rigorously evaluated against a panel of four radiologists. To our knowledge, this is the first study to assess the diagnostic utility of baseline CXRs for distinguishing COVID-19 from other viral pneumonias while benchmarking DL performance against multiple expert readers.
- *Robustness and generalizability assessment:* We assessed the framework’s robustness through subgroup analyses by age, sex, and disease severity, demonstrating consistent performance across diverse patient profiles. Additionally, external validation of both the diagnostic and severity scoring modules on publicly available datasets confirmed the model’s generalizability.
- *Open resource contribution:* The severity scoring model was evaluated on 86 publicly available COVID-19 CXRs annotated by an expert radiologist. The corresponding quadrant-level and overall severity scores will be made publicly accessible to support future research on automated severity assessment of pneumonia.

## Related works

AI-based solutions have been widely applied across different domains such as traffic prediction [23], cybersecurity [24], and energy-efficient wireless sensor networks [25], to name but a few. In healthcare, AI has advanced cardiac disease prediction [26], medical image enhancement [27], and automated diagnosis. The COVID-19 pandemic has accelerated the use of DL for pneumonia detection on chest X-rays, facilitated by the release of several public multi-center CXR repositories. While these datasets have enabled significant research progress, they often lack key clinical information such as the timing of image acquisition relative to disease progression and whether they represent the full spectrum of disease severity. Many DL models built on these datasets report high classification accuracy, but often without evaluating performance across severity levels, which is critical for clinical triage.

Despite promising results in pneumonia diagnosis from CXRs, the performance and reliability of DL models remain highly dependent on dataset quality. While heterogeneity across datasets can enhance generalization, variations in imaging protocols between centers may introduce bias. In one study involving four public CXR datasets, models achieved high COVID-19 classification accuracy even when lung regions were completely masked out [28], suggesting that predictions may have been driven by dataset-specific artifacts rather than clinically relevant features. Some studies address clinical relevance by evaluating model performance across varying severity levels or comparing predictions with radiologist interpretations. Tabik et al. [29], for example, created a balanced dataset of 426 COVID-positive and 426 negative CXRs, collected under a consistent protocol across the full spectrum of severity. Their ResNet-50-based model, enhanced with GAN-inspired augmentation, achieved an overall accuracy of 76.18% and F1-score of 75.71%. However, performance dropped for early-stage cases (Mild: 61.8%; Normal-PCR + : 28.42%), highlighting challenges in subtle radiographic presentations.

Wang et al. [11] proposed a pipeline combining anatomical landmark detection, lesion segmentation, and classification. Their model achieved an AUC of 0.966 for classifying COVID-19 versus other pneumonias (viral and bacterial), but performance declined to 0.867 when distinguishing COVID-19 from viral pneumonia alone, highlighting the challenge of differentiating between viral pathogens. Similarly, Miyazaki et al. [17] trained an EfficientNet-based model on a multi-center dataset of 26,393 CXRs to classify COVID-19, bacterial pneumonia, and normal cases. On an independent test set of 180 CXRs from a single center, the model achieved 0.733 accuracy, with 0.667 sensitivity for COVID-19 detection. A comparative analysis with radiologists showed that DL assistance improved their accuracy from 0.696 to 0.723 and increased COVID-19 sensitivity from 0.552 to 0.6.

A separate evaluation [20] of a commercial DL-based computer-aided diagnosis (CAD) system further demonstrated the value of AI assistance. Using a dataset of 172 CXRs (80 COVID-positive and 92 negative), the CAD system achieved an AUC of 0.714, comparable to thoracic radiologists and superior to non-radiologist physicians. CAD assistance significantly improved non-radiologist performance, increasing their AUC from 0.584 to 0.664 for COVID-19 detection and from 0.650 to 0.738 for pneumonia. The CAD system also showed increased sensitivity for patients with symptom onset beyond five days (90.3%) and those with CT severity scores above 10 (89.5%), but performance dropped for earlier-stage or less severe cases.

Building on ensemble approaches, [30] introduced a multi-stage DL framework consisting of lung segmentation, followed by classification through a fuzzy rank-based ensemble of three CNN models: DenseNet-201, SE-Inception-V3, and SE-SqueezeNet. Trained on public datasets in which COVID-19 images originated from a different source than the normal and pneumonia cases, the model achieved a high classification accuracy of 98.05%. However, most misclassifications occurred between pneumonia and normal classes, both sourced from the same dataset, suggesting that the model may have relied on center-specific features. Although many studies address COVID-19 diagnosis from CXRs, distinguishing COVID-19 from non-COVID-19 viral pneumonia, particularly using early-stage CXRs, remains relatively underexplored.

Beyond COVID-19 diagnosis, severity assessment from CXRs plays an important role in tracking disease progression, guiding clinical decisions, and managing resources. COVID-19 severity scoring on CXRs is mostly based on three key factors: the extent of lung involvement, the distribution of infection across lung regions, and opacity characteristics. DL models have employed various severity scoring systems with different strategies and scoring scales. BS-Net [14], for example, predicts Brixia (ranging from 0 to 18) using U-Net++ for segmentation, a transformer-based alignment module, and a multi-branch attention network. It achieved an MAE of 1.728 and a correlation of 0.862 with radiologist-provided scores.

Another study [15] proposed a GAN-based pipeline to synthesize CXR views from CT-derived images, estimating pneumonia extent as a percentage of lung area. The predictions moderately correlated with Brixia and CT-based scores and were prognostic across external datasets (AUC up to 0.842). COVID-Net S [31] predicted geographic and opacity extent scores as two separate scores, achieving R^2^ values of 0.739 and 0.741. In this study, we adopt a severity scoring approach inspired by the RALE score [32] for assessing pulmonary edema in acute respiratory distress syndrome (ARDS). Similar to RALE, our system incorporates both the extent of lung involvement and the type of radiographic opacities, evaluated across predefined lung regions. Table 1 presents a comparative summary of prior studies and the proposed DL framework.

**Table 1:** Comparative summary of prior studies and the proposed DL framework. CI: Confidence Interval; pn: pneumonia; CV: cross-validation.

Ref	Training Dataset	Model	CXR classes	Hold-out Validation	External Validation	Evaluation across			Compare with Rad.	COVID-19 vs Viral	Early CXR	Severity module
						Severity	Age	Gender				
[11]	Multi-center	CNN-based ensemble	COV-19, Viral pn, Bacterial pn, Normal	CI	✔	✔			✔	✔		✔
[17]	Multi-center	EfficientNet-based	COV-19, Bacterial pn, Normal	N.A.	✔				✔			
[20]	COV-19: multi-center, Normal: single-center	Commercial DL system	COV-19 vs Normal.	N.A.	✔	✔			✔		✔	
[21]	Multi-center	CNN model	COV-19; Viral Pn.; Normal.	Train/Test split						✔		
[22]	Multi-center	VGG16	COV-19; Viral Pn.; Normal.	Train/Test split						✔		
[29]	Single-center	ResNet-50-based	COV-19 pos. vs COV-19 neg.	5 times 5-fold CV		✔						
[30]	Multi-center	CNN-based ensemble	COVID-19, Other pn, Normal.	Train/Test split	✔	✔			✔			✔
[33]	Multi-center	ViT	COV-19; Viral Pn.; Lung Opacity; Normal.	Train/Test split						✔		
[34]	Multi-center	Tuned EfficientNet	COV-19; Lung Opacity; Viral Pn.; Normal.	Train/Test split						✔		
[35]	Multi-center	Tailored CNN	COV-19; Other Pn. (Bact. + Viral); Normal.	Train/Test split								
[36]	Multi-center	Capsule network-based	COV-19 Pos. vs COV-19 Neg.	Train/Test split								
Proposed system	Single-center	CNN-based framework	COV-19; Viral pn.; Normal.	5-fold CV + CI	✔	✔	✔	✔	✔	✔	✔	✔

## Materials and methods

### Data acquisition

**Fig 1:**
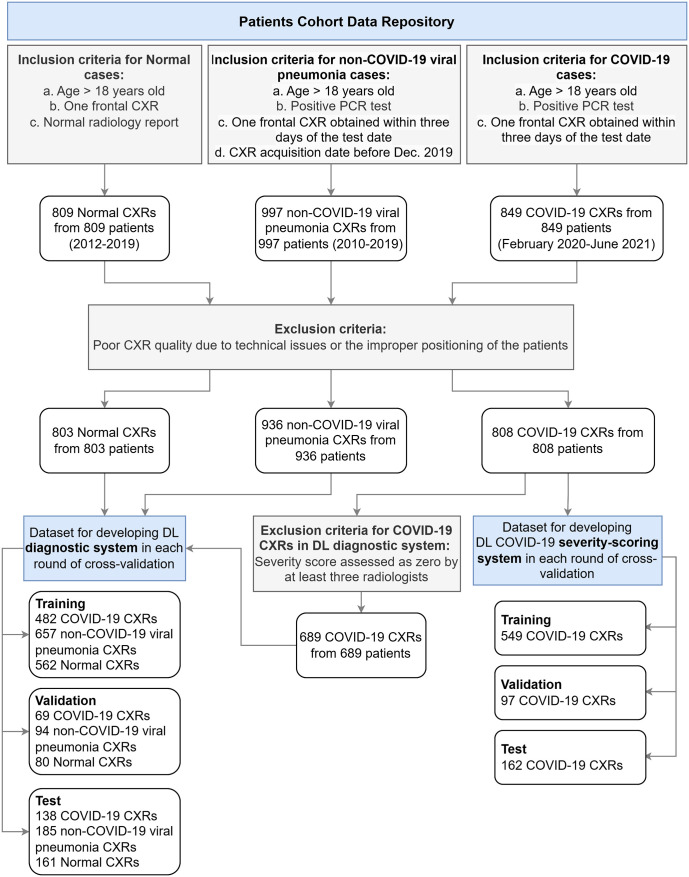
Diagram illustrating the patient cohort included in this study, showing the selection process for COVID-19, non-COVID-19 viral pneumonia, and normal cases.

This retrospective study was approved by our institution’s research ethics board (REB Project ID: 2153). Informed written consent was waived, and data were anonymized and collected in accordance with local guidelines. Consecutive CXRs were retrieved from our institution’s database, excluding patients under 18 years of age. Fig 1 illustrates the patient cohort selection process. Inclusion criteria for normal cases required one frontal CXR. For COVID-19 and non-COVID-19 viral pneumonia cases, inclusion required a positive PCR test and a frontal CXR obtained within three days of the test date. Non-COVID-19 viral pneumonia cases were further restricted to images acquired before December 2019, the onset of the pandemic.

The initial dataset included 841 COVID-19, 997 non-COVID-19 viral pneumonia cases (collected as part of two previous studies) [37,38], and 809 normal CXRs. After excluding images with poor quality due to technical issues or improper patient positioning, the final dataset included 808 COVID-19, 936 non-COVID-19 viral pneumonia (177 non-COVID-19 coronavirus and 759 other viral pneumonia), and 803 normal CXRs. COVID-19 CXRs were acquired between January 23, 2020, and June 3, 2021, from isolation wards of the emergency department, intensive care units, or through bedside imaging, following pre-approved local protocols. Non-COVID-19 viral pneumonia CXRs included seasonal non-COVID-19 coronaviruses collected between 2010 and 2016, and additional non-COVID-19 coronavirus and other viral infections collected between 2016 and 2019 [37]. Collecting these cases before the onset of the pandemic ensured that they were not COVID-19. Normal CXRs collected between 2012 and 2019 were identified based on radiology report conclusions containing phrases such as “Unremarkable study” or “No acute intrathoracic abnormality detected”, and confirmed with negative PCR results when available. The reference standard for COVID-19 and non-COVID-19 viral pneumonia cases was PCR positivity, and for normal cases, it was the radiology report.

### CXRs diagnosis and severity scoring by radiologists

All CXRs were interpreted independently in two phases by four staff radiologists: three fellowship-trained thoracic radiologists with 20, 12, and 8 years of experience, respectively, and one fellowship-trained emergency and trauma radiologist with 17 years of experience. In the diagnostic phase, the radiologists categorized each CXR as normal (0), non-COVID-19 viral pneumonia (1), or COVID-19 (2). Imaging findings in favor of COVID-19 included symmetric, bilateral, patchy, ill-defined peripheral opacities with mid and lower-zone predominance [8]. Imaging findings suggestive of non-COVID viral infection included predominantly central interstitial or peribronchial airspace opacities, the presence of reticulonodular or nodular patterns, and the unilateral distribution [39]. The majority vote was considered the consensus label. In cases with no majority vote, the highest voted category was assigned. During this phase, the readers were blinded to all patients’ clinical information to minimize bias and examine interobserver reliability.

In the severity scoring phase, the radiologists assessed baseline COVID-19 CXRs, being blinded to all patient data except for the PCR positivity. The average of the four radiologists’ scores was used as the consensus ground truth for DL model training. Severity scoring involved two main steps. First, each lung was divided into four quadrants on the frontal chest projection, vertically by the vertebral column and horizontally by the carina, labeled as Q1 (RUL: right upper lung zone), Q2 (RLL: right lower lung zone), Q3 (LUL: left upper lung zone), and Q4 (LLL: left lower lung zone). When the carina was not clearly visible, the lungs were divided into four equal zones.

Second, each quadrant was assessed for extent and density of lung involvement. The extent score ranged from 0 to 4 based on the percentage of opacification: 0 (normal), 1 (1–24%), 2 (25–49%), 3 (50–74%), and 4 (75–100%). The density score ranged from 0 to 3: 0 (normal), 1 (interstitial reticular), 2 (hazy/ground-glass opacity), and 3 (consolidation). If two patterns were present, the higher score was assigned. Radiographic features were diagnosed following the Fleischner Society glossary [40]: ground glass opacity (GGO) was characterized as increased lung opacification without obscure blood vessels and airways, consolidation was defined as homogeneous opacification that obscured vessels and airway walls, and reticulation was identified by the presence of innumerable small opacities in a linear pattern.

**Fig 2:**
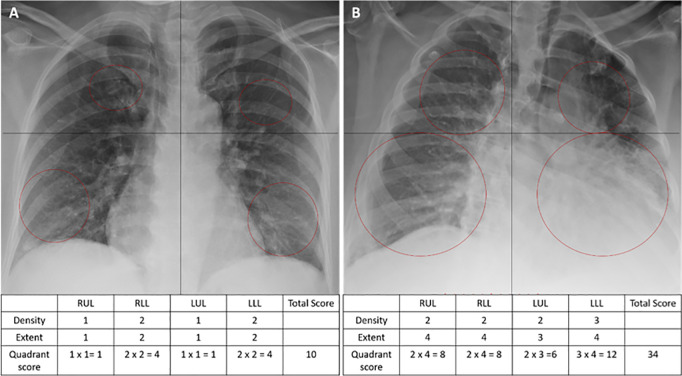
Examples of CXR severity score evaluations by two radiologists. The black vertical and horizontal lines indicate the four lung zones. Red circles highlight lung abnormalities. (A) CXR shows a mild reticular pattern in both upper lobes with an extent of <24% and ground glass opacities (GGO) in both lower lobes with an extent of 25−49% on each lobe. A total score of 10 was assigned by both readers. This patient was discharged home from the emergency department. (B) CXR shows more involvement of the lower lobes (75−100%), with consolidation in the left lower lobe. A total score of 34 was assigned by both readers. The patient passed away 23 days post COVID-19 test positivity.

Each quadrant’s severity score was calculated as the product of the extent and density scores (range 0–12). The sum of all quadrants yielded the total CXR severity score (range 0–48). Fig 2 provides examples of CXR severity scores assigned by two radiologists.

### Image preprocessing and augmentation

All CXR images were preprocessed using a standardized pipeline to ensure to ensure consistent input dimensions and intensity normalization. DICOM files were loaded using the SimpleITK library and converted to NumPy arrays. For images with the MONOCHROME1 photometric interpretation, pixel intensities were inverted to provide consistent contrast orientation over the dataaset. The original aspect ratios had a mean of 1.05 ± 0.14 (IQR: 0.97–1.20), therefore, all images were resized to a square format (512 × 512 pixels) using bilinear interpolation to maintain anatomical proportions with minimal distortion. Each image was converted to a three-channel format by replicating the grayscale image across the RGB channels and normalized using its own mean and standard deviation.

To further improve model generalization and reduce overfitting, real-time data augmentation was applied during training. The augmentation strategy included random rotations up to ±5°, horizontal and vertical shifts (up to 5% of the image dimensions), and zooming (±5%). Nearest-neighbor interpolation was used to fill gaps introduced by geometric transformations. Augmentations were applied to each mini-batch during training, producing new variations of the training images at each epoch. This approach effectively increases dataset variability, helping the model learn more robust features.

### DL system architecture

Deep convolutional neural networks (CNNs) have demonstrated exceptional capability in extracting informative features from medical images, making them the dominant architecture in medical image analysis. Our proposed DL framework is entirely CNN-based and consists of three key components. First, a thorax segmentation module processes frontal chest X-rays (both posteroanterior and anteroposterior views) to generate thorax region masks. These masks are used to extract the thoracic area from the original images. The extracted thorax regions from the baseline CXRs are then fed into a diagnostic module that classifies them as COVID-19, other viral pneumonia, or normal. Cases predicted as COVID-19 are further analyzed by a severity scoring module, which estimates a severity score based on the extent and pattern of lung involvement. To improve model sensitivity to infection-related features, the framework leverages a hierarchical transfer learning strategy to enhance learning from limited medical data. The following sections describe each component of the framework in detail.

**Fig 3:**
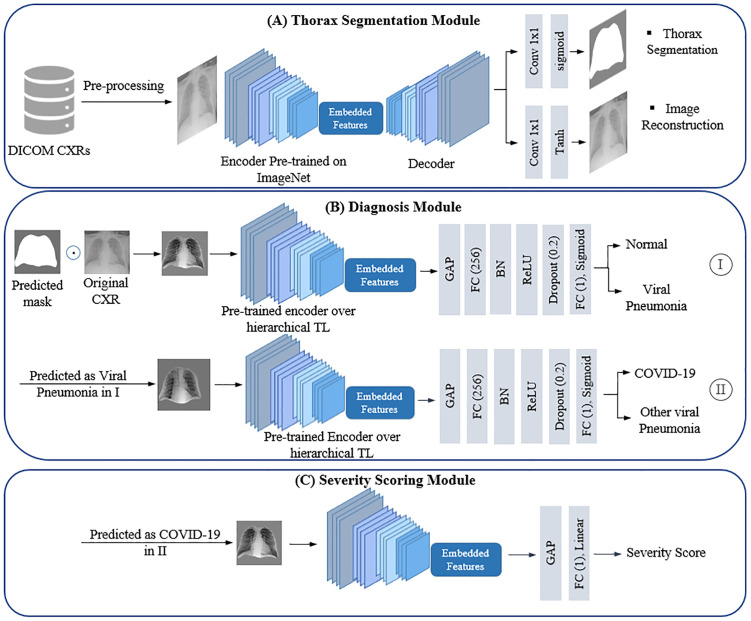
The proposed DL system pipeline. (A) Thorax segmentation module: Predicts thorax region masks for each CXR using a multi-task learning approach. The thorax masks are used to extract thorax regions from the original CXRs, which serve as inputs to the subsequent modules. (B) The diagnosis module. I: Diagnosing viral pneumonia cases from normal ones. II: Receiving cases predicted as viral pneumonia in step one to classify them as COVID-19 or other viral pneumonia. (C) The severity scoring module: Estimates disease severity from the input CXRs.

- **Thorax segmentation module:** To explicitly constrain the model’s attention to relevant anatomical regions and minimize background noise, we developed a thorax segmentation module, as shown in Fig 3A, based on a U-Net architecture [41] with an InceptionResNet-v2 encoder backbone [42] pre-trained on the ImageNet dataset. The architecture consists of an encoder that leverages the deep residual and multi-scale feature extraction capabilities of InceptionResNet-v2, while the decoder follows the standard U-Net upsampling and skip-connection pattern to enable precise localization. The segmentation network outputs a thorax mask, which is subsequently applied to extract the region of interest (ROI) for downstream diagnostic modules, ensuring that all further analysis is restricted to the thoracic regions.

Unlike conventional lung-only segmentation approaches, we designed the thorax mask to encompass the entire thoracic region, including mediastinal and central structures, to preserve diagnostically significant features near the heart that may otherwise be excluded. The model was trained on a curated set of 316 CXRs (150 COVID-19 and 166 non-COVID viral pneumonia cases), with manually annotated thorax masks provided by one of our four radiologists (Reader 2).

To further enhance feature learning in the limited-data regime, we employed a multi-task learning (MTL) strategy [43], where the model simultaneously optimizes for thorax segmentation (pixel-wise classification) and CXR reconstruction (image-to-image mapping). This design allowed the encoder to learn richer features by leveraging complementary information from both tasks, thereby improving generalization performance with limited annotations.

The segmentation head outputs a 512 × 512 binary mask, activated via Sigmoid, while the reconstruction head produces a 512 × 512 × 1 image, normalized with Tanh activation to enforce pixel intensity consistency in the range [–1, 1].

For the segmentation task, which involved pixel-level binary classification, we used a combination of Binary Cross-Entropy and Dice loss. Mean Squared Error was employed for the image reconstruction task. The total loss (L_total_) used to train the multi-task learning (MTL) network was computed as a weighted sum of the segmentation loss (L_seg_) and the reconstruction loss (L_recon)_.


$$L_{total}=\;(1\;-\;\gamma )\cdot L_{seg}+\;\gamma \cdot L_{recon},\;with\;\gamma =\;0.1$$ (1)

The segmentation loss combines Binary Cross-Entropy with Dice loss to handle class imbalance and enforce region-level consistency. The reconstruction task serves as an auxiliary supervision signal, enhancing encoder robustness and promoting better generalization. The loss weighting factor γ was empirically set to 0.1, emphasizing segmentation while still guiding the network to preserve global structure.

**Fig 4:**
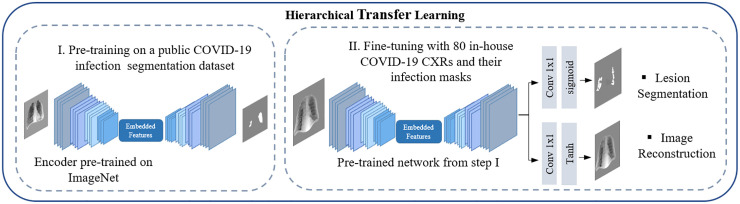
Hierarchical transfer learning process. Step I: Pre-training on a public COVID-19 segmentation dataset. Step II: Fine-tuning using a subset of 80 COVID-19 CXRs from our in-house dataset. The pre-trained encoder was used as the foundation of the diagnosis and severity scoring modules.

- **Hierarchical transfer learning:** We employed a hierarchical transfer learning process to enhance the model’s focus on infection regions, as illustrated in Fig 4, Building on a U-Net segmentation network with an InceptionResNet-v2 encoder backbone pre-trained on ImageNet, we structured the training in two distinct phases. In the first phase, all network layers were unfrozen to enable full weight adaptation using 2,000 COVID-19 CXRs with corresponding lesion masks from a publicly available repository [44]. This phase established initial feature representations for infection segmentation by leveraging a large, diverse dataset. In the second phase, the model was fine-tuned on a subset of 80 COVID-19 CXRs from our in-house dataset, with infection masks carefully annotated by a radiologist (Reader 2).

To further enhance learning during the second phase, we adopted a multi-task learning approach that enabled the model to perform both infection segmentation and image reconstruction simultaneously, promoting feature learning from a limited dataset. In both phases, the model received thoracic regions extracted from CXRs as input, with the data split into 80% for training and 20% for validation. By combining large-scale initial training with specialized fine-tuning and multi-task learning, this hierarchical approach enabled the model to refine its understanding of infection patterns within the thoracic region.

- **Diagnosis and severity scoring modules:** The pre-trained encoder of the segmentation network from the last phase of transfer learning served as the foundation for both diagnostic and severity scoring modules. By leveraging this encoder, we were able to transfer learned representations of infection-specific features for enhanced accuracy in downstream tasks. Both modules receive a 16×16×1536 feature map from the InceptionResNet-v2 encoder. A Global Average Pooling (GAP) layer is applied after the encoder to reduce this spatial representation into a 1×1536 vector by averaging across each feature map.

In the diagnostic model, as shown in Fig 3B, this vector is passed through a fully connected (FC) layer with 256 neurons, followed by batch normalization (BN) to stabilize learning by normalizing activations and reducing internal covariate shift. A rectified linear unit (ReLU) activation introduces non-linearity, and a Dropout layer (rate = 0.2) is applied to randomly deactivate neurons during training and reduce overfitting. The final output layer is a single-neuron dense layer with a Sigmoid activation, producing a probability score for binary classification. The diagnostic model is trained in two stages: first to differentiate viral pneumonia from normal cases, and then to distinguish COVID-19 from other types of viral pneumonia.

For the severity scoring module, the same 1 × 1536 vector from the GAP layer is passed directly into a single-neuron fully connected layer with a Linear activation, generating a continuous severity score ranging from 0 to 48. The detailed structure of the severity scoring module is shown in Fig 3C.

### Model training and implementation setup

During the training phase, the model weights were updated iteratively based on the loss calculated from each mini-batch of the training data. To prevent overfitting and information leakage, a separate validation set was used to tune hyperparameters and monitor model performance throughout training. Early stopping was employed to terminate training if no improvement was observed on the validation set for 10 epochs. The final model was selected based on its best validation performance. The test set was reserved exclusively for final evaluation after training, ensuring that the model development was not influenced by the test outcomes.

All experiments were conducted on a local workstation running 64-bit Windows 10, equipped with an Intel Core i9 processor (4.48 GHz), 64 GB of RAM, and an NVIDIA GeForce RTX 3080 GPU with 11 GB of dedicated memory. Models were developed in Python 3.9.13 using Keras 2.10.0, TensorFlow 2.10.0, and standard scientific libraries including NumPy 1.26.4, SciPy 1.13.1, OpenCV 4.5.5, SimpleITK 2.2.1, and scikit-learn 1.2.2. GPU acceleration was enabled via CUDA 11.2 and cuDNN 8.

U-Net and its variants were implemented using the “segmentation_models” library, which offers pre-built architectures with ImageNet-pretrained encoders and supporting utilities. Training time was approximately 1 hour for the segmentation model and 1–2 hours for the classification and severity scoring models. The average inference time per CXR was approximately 62 milliseconds for thorax segmentation, 58 ms for the two-step classification module, and 24 ms for the severity scoring module.

### Statistical analysis

We employed 5-fold cross-validation to train and validate the DL system. For the diagnostic module, stratified cross-validation was used to maintain consistent class distribution across all training, validation, and test sets. Confidence intervals (CIs) for the diagnostic performance metrics were calculated to evaluate the statistical robustness of the model across variations in class distributions and demographic subgroups. The model was independently trained eight times using different randomized train/test splits, with 80% of the data used for training and validation and the remaining 20% held out for testing. 200 bootstrap resampling iterations with replacement were performed on each test set, resulting in 1,600 performance estimates. CIs were derived from the distribution of these evaluations.

The McNemar-Bowker test was applied to the same eight independent test sets to assess whether there were statistically significant differences between the DL model’s and the radiologists’ diagnostic performance. Inter-rater agreement between radiologists in the diagnosis task was assessed using Fleiss’ Kappa (ĸ) [45], which evaluates consistency in categorical ratings across multiple raters. For severity scoring, the Intraclass Correlation Coefficient (ICC) was used to assess agreement among radiologists. Specifically, we used ICC(2,k) [46], which assumes both CXRs (subjects) and radiologists (raters) are random samples from a broader population, and accounts for potential systematic differences in scoring.

### Evaluation metrics

We utilized standard evaluation metrics to assess the performance of the thorax segmentation, classification, and severity scoring modules. For segmentation, we used the Dice Similarity Coefficient (DSC) and Intersection over Union (IoU). DSC quantifies the overlap between predicted and ground truth masks, ranging from 0 to 1, where 1 indicates perfect agreement. IoU similarly measures the ratio of the intersection to the union of the predicted and ground truth masks, with higher values indicating better alignment. For the diagnostic module, we used recall, positive predictive value (PPV), F1-score, accuracy, and the area under the receiver operating characteristic curve (AUC). Recall measures the proportion of correctly identified positive cases, while PPV calculates the proportion of true positives among all positive predictions. F1-score, the harmonic mean of recall and PPV, balances false positives and false negatives. AUC was computed to assess the overall discrimination ability of the model across both stages of the diagnostic pipeline.

For the severity scoring module, we used Pearson correlation coefficient and Mean Absolute Error (MAE). Pearson correlation measures the strength of the linear relationship between predicted and radiologists’ scores, while MAE reflects the average magnitude of prediction errors. Metric definitions are provided below, where TP, FP, TN, and FN represent true/false positives and negatives. For Pearson correlation and MAE, $x_{i}$ and $y_{i}$ are the ground-truth and predicted severity scores for the i^th^ sample, $\bar{x}$ and $\bar{y}$ are their respective means, and n is total number of samples.


$$DSC\;=\;\frac{2\;\times \;TP}{\left(2\;\times \;TP\;+\;FP\;+\;FN\right)}$$ (2)

$$IoU\;=\;\frac{TP}{\left(TP\;+\;FP\;+\;FN\right)}$$ (3)

$$Recall\;=\;\frac{TP}{\left(TP\;+\;FN\right)}$$ (4)

$$PPV\;(Precision)=\;\frac{TP}{\left(TP\;+\;FP\right)}$$ (5)

$$F1-Score\;=\;2\;\times \frac{\left(Precision\;\times \;Recall\right)}{\left(Precision\;+\;Recall\right)}$$ (6)

$$Accuracy\;=\frac{\left(TP\;+\;TN\right)}{\left(TP\;+\;TN\;+\;FP\;+\;FN\right)}$$ (7)

$$\text{Pearson\textbackslash{} correlation\textbackslash{} coefficient }=\frac{\Sigma \left[\left(x_{i}-\;\bar{x}\right)\left(y_{i}-\bar{y}\right)\right]}{\left[\sqrt{\Sigma {\left(x_{i}-\;\bar{x}\right)}^{2}}\times \;\sqrt{\Sigma {\left(y_{i}-\bar{y}\right)}^{2}}\right]}$$ (8)

$$MAE\;=\;\left(\frac{1}{n}\right)\times \Sigma \left|y_{i}\;-\;x_{i}\right|$$ (9)

## Results

### Patient characteristics

**Fig 5:**
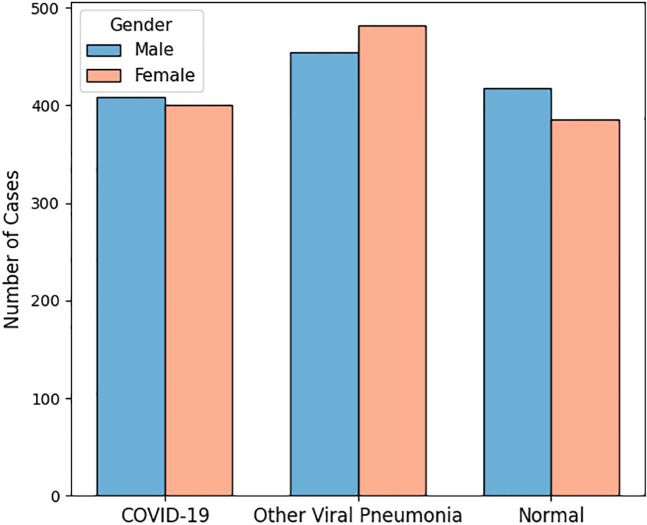
Number of male and female patients across the three diagnostic groups.

**Fig 6:**
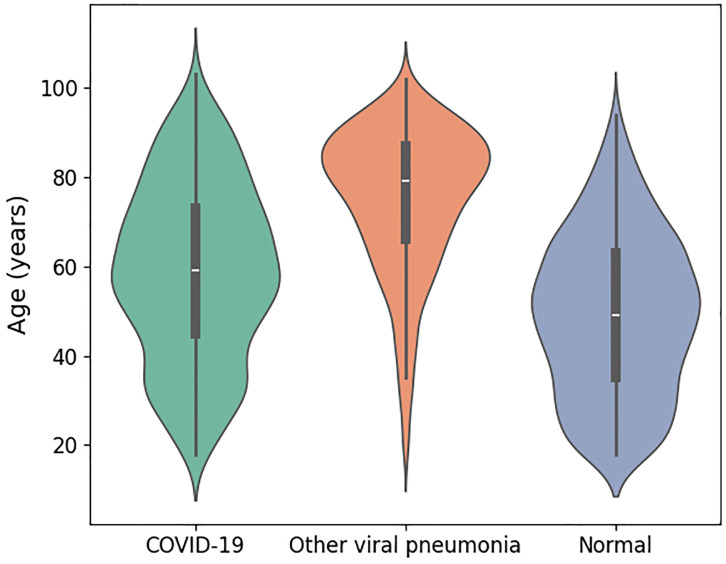
Age distribution of patients across the three diagnostic groups.

After excluding poor quality images, a total of 2,547 CXRs were included in the analysis: 808 COVID-19, 936 non-COVID-19 viral pneumonia, and 803 normal CXRs. Table 2 summarizes the demographic characteristics of patients across these three groups. Gender distribution is shown in Fig 5, indicating a relatively balanced number of male and female patients in each category. Fig 6 illustrates key statistical characteristics of age distributions in each class. As illustrated, normal cases are mostly concentrated in younger individuals, while other viral pneumonia cases span a wider range and are skewed toward older individuals. COVID-19 cases show a more even and moderately wide age distribution. Table 3 represents the distribution of different non-SARS-Cov-2 viral infections in the dataset.

**Table 2:** Demographic characteristics of the study population across diagnostic groups, including the number of CXRs, mean patient age ± standard deviation, and percentage of female patients in each group.

	COVID-19	non-COVID-19 Viral Pneumonia		Normal
		Coronavirus	Other types of virus	
No. of CXRs	808	177	759	803
Age	59 ± 20	74 ± 17	75 ± 16	49 ± 17
Female (%)	49.5	51.1	51.8	47.9

**Table 3:** Number of different types of Non-SARS-Cov-2 viruses.

Virus type (collected between 2010–2019)	#Number
01. H1N1	53 (5.3%)
02. H3N2	192 (19.3%)
03. FluB	123 (12.3%)
04. RSV	105 (10.5%)
05. PIV	37 (3.7%)
06. Rhino	124 (12.4%)
07. CoV	245 (24.6%)
• 229E	• 40 (4%)
• NL63	• 42 (4.2%)
• OC43	• 135 (13.5%)
• HKU1	• 28 (2.8%)
08. hMPV	94 (9.4%)
09. ADV	3 (0.3%)
10. mixed	8 (0.8%)
11. others	13 (1.3%)
Total	997

**Fig 7:**
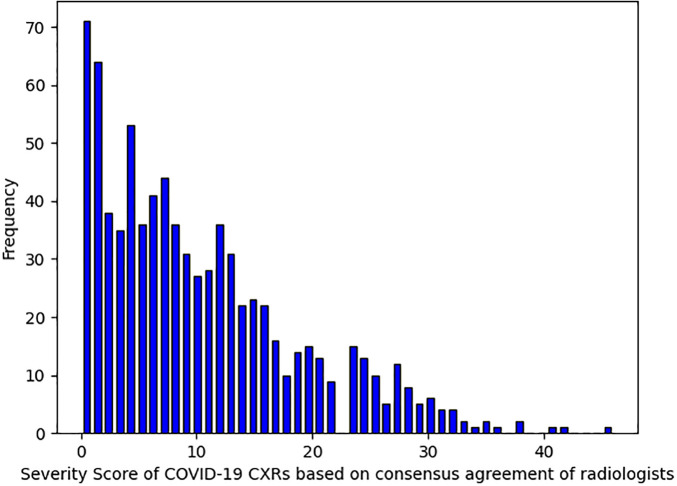
The distribution of severity scores in our COVID-19 CXRs based on the average evaluations of four radiologists.

Given the focus on baseline CXRs, it is notable that a significant portion of COVID-19 images showed minimal or no radiographic signs of infection. As shown in Fig 7, approximately 33% of COVID-19 cases received an average severity score of four or lower, and 15% received a score of zero from at least three out of four radiologists. In contrast, although the severity scores of non-COVID-19 viral infection cases were not quantified, we did not observe a substantial number of normal-appearing CXRs in this group. To fairly assess the diagnostic utility of baseline CXRs in distinguishing COVID-19 from other viral infections, we defined two diagnostic scenarios. The first scenario excluded COVID-19 CXRs with a severity score of zero according to at least three radiologists, leaving 689 COVID-19 CXRs for model training and evaluation. Each cross-validation round included 1,701 CXRs for training (482 COVID-19, 657 non-COVID-19 pneumonia, 562 normal), 243 CXRs for validation (69 COVID-19, 94 non-COVID-19 pneumonia, 80 normal), and 484 CXRs for testing (138 COVID-19, 185 non-COVID-19 pneumonia, 161 normal). In the second scenario, all 808 COVID-19 CXRs, including those without visible abnormalities, were used to evaluate diagnostic performance.

The complete set of 808 COVID-19 images was also used for training and testing the DL severity scoring module. Each cross-validation round included 549 COVID-19 CXRs for training, 97 for validation, and 162 for testing, respectively.

### Hyperparameter tuning

We systematically tuned key hyperparameters to optimize model performance, focusing on the initial learning rate, batch size, and dropout rate. The results, shown in Table 4, correspond to the first step of the diagnostic module. We tested a range of learning rates across three repeated runs using the same data splits. Based on accuracy, F1-score, and consistency, 0.0001 and 0.0005 were shortlisted and further assessed across three randomized train/test splits. A learning rate of 0.0005 yielded the best overall performance and stability and was selected for the final model. All models were trained using the Adam optimizer with a learning rate decay of 0.0004 to gradually reduce the learning rate during training. The same tuning process was applied to the second diagnostic step and the severity scoring module, in which a learning rate of 0.0001 yielded the best results.

**Table 4:** Performance metrics for hyperparameter tuning of the diagnostic module. Results are reported as mean ± standard deviation over three runs. “*” indicates experiments repeated with the same data split, while “**” indicates runs with three randomized train/test splits.

Hyperparameter	Value	Recall (%)	Precision (%)	F1-score (%)	Accuracy (%)
Learning Rate *	0.00001	88.9 ± 5.1	89.2 ± 3.1	88.9 ± 1.9	85.5 ± 2.2
	0.00005	90.1 ± 2.7	89.8 ± 1.9	89.9 ± 1.3	86.8 ± 1.6
	0.0001	93.9 ± 1.3	86.6 ± 0.4	90.1 ± 0.4	86.5 ± 0.4
	0.0005	89.9 ± 1.2	91.6 ± 0.7	90.7 ± 0.4	87.9 ± 0.5
	0.001	94.5 ± 1.9	86.1 ± 4.6	90.0 ± 1.9	86.2 ± 3.2
Learning Rate **	0.0001	88.3 ± 5.2	89.6 ± 4.1	88.7 ± 1.1	85.4 ± 1.0
	0.0005	87.9 ± 1.8	90.7 ± 0.4	89.3 ± 1.0	86.2 ± 1.2
Batch Size **	2	87.8 ± 1.0	87.4 ± 3.2	87.6 ± 1.3	83.6 ± 2.1
	4	87.9 ± 1.8	90.7 ± 0.4	89.3 ± 1.0	86.2 ± 1.2
	8	86.8 ± 5.3	90.0 ± 5.4	88.2 ± 1.4	84.7 ± 1.9
Dropout Rate **	0.1	86.2 ± 1.6	89.4 ± 1.1	87.8 ± 1.3	84.2 ± 1.7
	0.2	87.9 ± 1.8	90.7 ± 0.4	89.3 ± 1.0	86.2 ± 1.2
	0.3	85.5 ± 7.2	91.8 ± 4.0	88.3 ± 1.9	85.2 ± 1.5

To assess the effect of batch size, we tested values of 2, 4, and 8 across three randomized splits. A batch size of 4 achieved the highest F1-score (0.893 ± 0.010) and accuracy (0.862 ± 0.012) with low variability and was therefore selected. We also tuned the dropout rate applied to the dense classification layer by testing values of 0.1, 0.2, and 0.3. A dropout rate of 0.2 consistently provided the best trade-off between performance and regularization and was used in the final models.

### Thorax segmentation performance

We evaluated several U-Net architectures for thorax segmentation using a dataset of 316 CXRs (150 COVID-19 and 166 other viral pneumonia cases), split into training (n = 220), validation (n = 60), and testing (n = 36) sets. Performance was averaged over four randomized splits and reported using Dice and IoU metrics.

**Fig 8:**
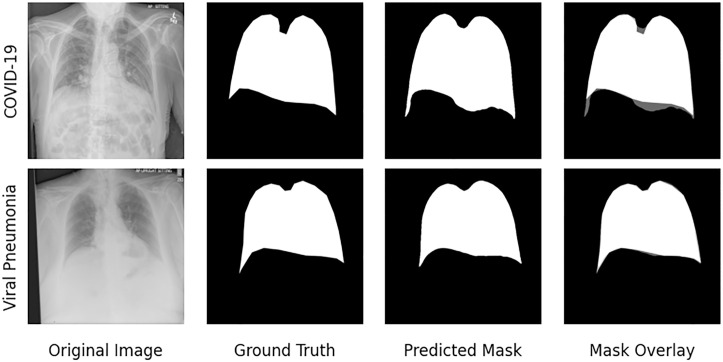
Examples of thorax segmentation results for COVID-19 and other viral pneumonia CXRs on th hold test set, with columns displaying the original chest X-ray, ground truth mask, predicted thorax mask, and the overlay of the predicted mask on the original image.

U-Net models with different ImageNet-pretrained backbones were tested, including VGG19, DenseNet121, ResNet50, and InceptionResNetV2. As presented in Table 5, InceptionResNetV2 achieved the highest performance among single-task models (Dice: 0.960 ± 0.008, IoU: 0.923 ± 0.015), slightly outperforming the others. We then extended this model to a multi-task architecture that jointly learned thorax segmentation and CXR reconstruction. This multi-task U-Net further improved performance (Dice: 0.964 ± 0.006, IoU: 0.931 ± 0.012), indicating that auxiliary reconstruction helps improve segmentation quality, especially in data-limited settings. Segmentation results for representative COVID-19 and viral pneumonia cases are illustrated in Fig 8, demonstrating high agreement between predictions and ground truth.

**Table 5:** Performance comparison of U-Net models with different backbone architectures for thorax segmentation. Results are reported as mean ± standard deviation of Dice and IoU scores across four randomized splits. MT: multi-task.

Model	IoU	Dice
UNet + Vgg19	0.916 ± 0.013	0.956 ± 0.007
UNet + Dens121	0.917 ± 0.017	0.957 ± 0.009
UNet + Resnet50	0.917 ± 0.011	0.957 ± 0.006
UNet + InceptionResV2	0.923 ± 0.015	0.960 ± 0.008
MT + UNet + InceptionResV2	0.931 ± 0.012	0.964 ± 0.006

To evaluate generalizability, the final segmentation model was applied to an external public dataset of 852 CXRs [29] (426 normal and 426 COVID-19 cases). Thorax masks were visually overlaid on the original images, and one radiologist reviewed a random subset, reporting accurate thoracic delineation in approximately 95% of cases.

### Diagnostic performance of the DL system and radiologists

The diagnostic module utilizes the thorax regions segmented from raw CXRs as input to a two-stage classification process. The first stage detects pneumonia CXRs from normal ones, and the second stage takes the images labeled as pneumonia to diagnose them as COVID-19 and other viral pneumonia.

**Fig 9:**
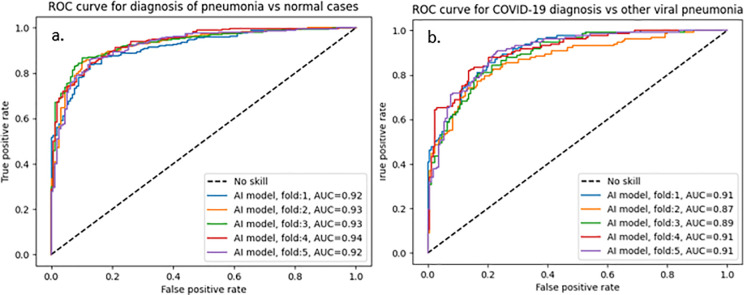
Receiver operating characteristic (ROC) curves of the two-stage DL diagnostic system across five-fold cross-validation. (a) ROC curves of stage one (diagnosis of pneumonia CXRs vs. normal CXRs). (b) ROC curves of stage two (diagnosis of COVID-19 CXRs vs. other viral pneumonia CXRs). AUC (Area Under the Curve) quantifies the overall diagnostic performance for each stage.

Fig 9 presents the ROC curves for both stages, showing AUCs of 93.0% ± 0.5 for pneumonia vs. normal and 89.8% ± 1.9 for COVID-19 vs. other viral pneumonia across five-fold cross-validation. Table 6 demonstrates performance metrics for individual readers, their consensus, and the DL system for the first scenario. The DL model outperforms the radiologists’ consensus and three out of four individual radiologists, achieving an accuracy of 76.4% (± 1.5), with F1 scores of 74.1% (± 1.5) for COVID-19, 74.2% (± 3) for non-COVID-19 viral pneumonia, and 80.7% (± 0.9) for normal cases. In comparison, the radiologists’ consensus achieved 71.8% (± 1.4) accuracy, with lower F1-score of 61.3% (± 3.3) for COVID-19, 69.3% (± 2.2) for non-COVID-19 viral pneumonia, and 79.2% (± 1.2) for normal cases. Individual radiologists’ performance showed significant variability, with COVID-19 F1 scores ranging from 30.4% to 78.9% and accuracies from 59.1% to 78.6%.

**Table 6:** The performance of individual radiologists, their consensus, and the DL model in the first diagnostic scenario (excluding COVID-19 CXRs with a severity score of zero as assessed by at least three radiologists).

Reader ID.	COVID-19			Other viral pneumonia			Normal			Accuracy (%)
	Recall (%)	PPV (%)	F1-score (%)	Recall (%)	PPV (%)	F1-score (%)	Recall (%)	PPV (%)	F1-score (%)	
1	18 ± 2.1	98.5 ± 3.3	30.4 ± 3.1	55.9 ± 3.2	57.4 ± 2.3	56.6 ± 2.6	97.3 ± 1.1	56.8 ± 0.9	71.7 ± 0.9	59.1 ± 1.4
2	74.1 ± 3.1	84.6 ± 3.5	78.9 ± 2.2	76.4 ± 2	74.4 ± 1.5	75.4 ± 1.3	84.8 ± 1.1	78.9 ± 1.9	81.8 ± 1.3	78.6 ± 0.8
3	51.7 ± 6.6	85.6 ± 1.7	64.3 ± 4.9	61.2 ± 1.1	74.5 ± 4.7	67.1 ± 2.1	98.3 ± 1.2	64.6 1.4	78 ± 1.4	71.2 ± 2.1
4	31.3 ± 2.6	78.6 ± 6.3	44.7 ± 2.6	78.2 ± 3.2	58 ± 1.9	66.6 ± 2	86.6 ± 2.7	76.6 ± 0.9	81.3 ± 0.8	67.5 ± 1.4
Readers Consensus	46 ± 4	92.3 ± 2	61.3 ± 3.3	67.3 ± 2.6	71.4 ± 3.1	69.3 ± 2.2	98.2 ± 0.5	66.3 ± 1.5	79.2 ± 1.2	71.8 ± 1.4
DL	74.6 ± 3.4	73.8 ± 3.2	74.1 ± 1.5	73.3 ± 5.6	75.5 ± 3.2	74.2 ± 3	81.4 ± 3.2	80.1 ± 2.6	80.7 ± 0.9	76.4 ± 1.5

To assess statistical reliability, we calculated 95% CIs for accuracy and F1-scores (Table 7). The DL system consistently outperformed Rads 1, 3, 4, and the consensus across all classes. For instance, in COVID-19 detection, the model achieved an F1-score of 73.4% (95% CI: 65.6–80.7), exceeding those of Rads 1, 3, 4, and the consensus. Rad 2 had the highest overall performance, with 78.9% (95% CI: 74.0–83.4) accuracy, outperforming the DL model and other readers. These confidence intervals highlight the expected variability of performance and support the potential clinical utility of the DL system.

**Table 7:** F1-scores and accuracy (95% CI) for individual radiologists, consensus, and the DL model.

Reader ID	F1-score COVID-19	F1-score Other Viral Pneumonia	F1-score Normal	Accuracy
1	30.5 (95% CI: 20.6–40.2)	57.2 (95% CI: 49.4–64.6)	71.8 (95% CI: 65.5–78.1)	59.6 (95% CI: 53.9–65.7)
2	79.1 (95% CI: 70.8–86.1)	75.8 (95% CI: 69.3–81.8)	81.9 (95% CI: 76.8–86.7)	78.9 (95% CI: 74.0–83.4)
3	63.4 (95% CI: 54.0–72.0)	66.2 (95% CI: 58.9–73.1)	77.2 (95% CI: 70.6–83.7)	70.4 (95% CI: 65.3–75.5)
4	42.5 (95% CI: 31.2–53.4)	66.6 (95% CI: 61.2–72.1)	81.8 (95% CI: 76.4–86.9)	67.6 (95% CI: 62.7–72.7)
Readers consensus	61.1 (95% CI: 50.9–70.7)	69.2 (95% CI: 62.6–74.9)	78.6 (95% CI: 72.8–84.2)	71.6 (95% CI: 66.6–76.6)
DL	73.4 (95% CI: 65.6–80.7)	74.6 (95% CI: 67.3–80.3)	80.5 (95% CI: 72.6–92.1)	76.4 (95% CI: 71.6–83.2)

McNemar-Bowker results across eight test sets confirmed the DL model significantly outperformed Rads 1, 3, 4, and the consensus in all experiments (*p < 0.0001*). However, Rad 2 outperformed the DL system in seven of eight sets (*p < 0.0005*), with no significant difference in the eighth (*p* *=* *0.28*).

**Fig 10:**
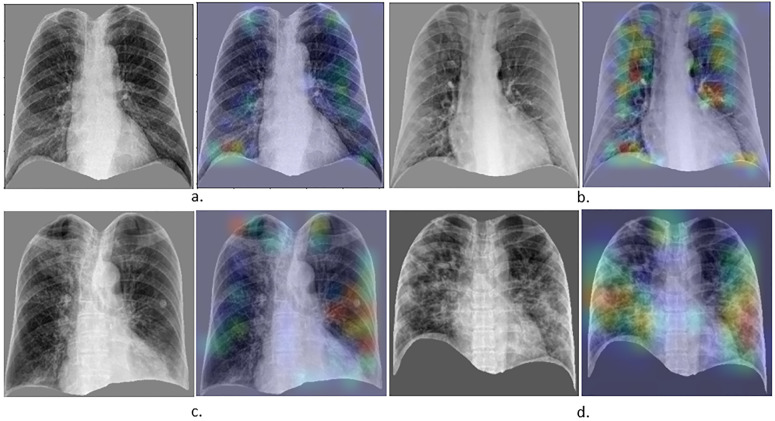
Frontal CXRs and corresponding Grad-CAM heatmaps for the two-stage DL diagnosis system. Top row: stage one (diagnosis of viral pneumonia vs. normal CXRs); (a) normal CXR (P = 0.24), (b) other viral pneumonia CXR (P = 0.99). Bottom row: stage two (diagnosis of COVID-19 vs. other viral pneumonia); (c) other viral pneumonia CXR (P = 0.03). (d) COVID-19 CXR (P = 0.99). P denotes the probability assigned by the DL model that a test CXR belongs to the target class.

The moderate inter-rater agreement among radiologists, reflected by a Fleiss’ Kappa of 0.52 [45], highlights interpretation variability. To further interpret model decisions, we used Grad-CAM [47], which revealed that the disease-affected regions within the lungs contribute the most to the model’s decision (Fig 10).

Table 8 compares the performance of the DL system and radiologists in the second scenario when all 808 baseline COVID-19 CXRs are included. The DL model outperformed the radiologists’ consensus and three individual readers. While radiologists demonstrated high accuracy in identifying normal CXRs (recall: 98.2% ± 1.7), the absence of findings in many COVID-19 cases led to frequent misclassification of COVID-19 CXRs as normal, resulting in a COVID-19 recall of 39% ± 6.6 and a PPV of 60.4% ± 3.6 for the normal cases. Including all COVID-19 CXRs, regardless of radiographic findings, led to a decrease in overall diagnostic performance. Nonetheless, the DL model achieved a higher accuracy (72.1% ± 2.5), compared to the radiologists’ consensus (68.2% ± 2.1).

**Table 8:** The performance of individual radiologists, their consensus, and the DL model in the second diagnostic scenario (including all 808 COVID-19 cases).

Reader ID.	COVID-19			non-COVID-19 viral pneumonia			Normal			Accuracy (%)
	Recall (%)	PPV (%)	F1-score (%)	Recall (%)	PPV (%)	F1-score (%)	Recall (%)	PPV (%)	F1-score (%)	
1	15.3 ± 2.4	98.4 ± 2.3	26.4 ± 3.6	55.9 ± 4.5	57.3 ± 2.3	56.6 ± 3.2	97.3 ± 1.6	52.2 ± 2	67.9 ± 1.8	56.3 ± 2.1
2	63.3 ± 3.6	84.6 ± 2.2	72.4 ± 3	76.4 ± 3.7	73.9 ± 1.5	75.1 ± 1.9	84.8 ± 1.1	70.0 ± 3.2	76.7 ± 1.6	74.9 ± 1.6
3	43.8 ± 6	85.3 ± 5.3	57.8 ± 6.3	61.2 ± 7.1	74.2 ± 2.8	66.8 ± 4.2	98.3 ± 0.3	59.1 ± 3.9	73.7 ± 3.1	67.7 ± 3.1
4	26.5 ± 4.6	78.3 ± 7.2	39.5 ± 5.4	78.1 ± 4.3	57.3 ± 2.3	66 ± 2.3	86.6 ± 2.3	68.7 ± 3.7	76.6 ± 2.5	64.2 ± 1.1
Readers Consensus	39.0 ± 6.6	91.9 ± 2.3	54.5 ± 6.6	67.3 ± 6.5	71.3 ± 1.9	69.1 ± 2.9	98.2 ± 1.7	60.4 ± 3.6	74.8 ± 2.8	68.2 ± 2.1
DL	62.2 ± 6.4	70.8 ± 4.7	66 ± 3.9	75.4 ± 7.5	73.2 ± 5.3	74 ± 4.2	78.3 ± 8.8	73.1 ± 5.3	75.3 ± 4.9	72.1 ± 2.5

#### Sensitivity analysis across patient subgroups.

We conducted a sensitivity analysis to evaluate the diagnostic model’s performance across key demographic subgroups, including age, sex, and disease severity, to assess potential biases. For age-based evaluation, the model achieved an AUC of 0.891 (95% CI: 0.826–0.939) in patients under 65 and 0.905 (95% CI: 0.827–0.960) for those aged 65 and older in step 1 (normal vs. pneumonia). In step 2 (COVID-19 vs. other viral pneumonia), performance was higher in the younger cohort (AUC: 0.930, 95% CI: 0.85–0.98) than in the older group (AUC: 0.849, 95% CI: 0.77–0.94), which might be attributed to greater imaging variability or overlapping features among older adults. Despite this moderate decline, the model maintained clinically meaningful performance across both age groups.

In the sex-based analysis, performance was comparable between females and males. In step 1, AUCs were 0.929 (95% CI: 0.88–0.97) and 0.922 (95% CI: 0.88–0.96), and in step 2, 0.904 (95% CI: 0.83–0.97) and 0.887 (95% CI: 0.82–0.94), respectively.

Table 9 presents the COVID-19 sensitivity of the DL model and radiologists across different severity levels. Severity was categorized as low (severity score ≤ 8), medium (8 < severity score ≤ 16), and severe (severity score > 16). Results, averaged over eight randomized splits, are reported as mean ± standard deviation.

**Table 9:** COVID-19 sensitivity (%) of the proposed DL diagnostic model compared to four individual radiologists and their consensus, stratified by severity level. Severity was categorized based on radiographic severity scores: low (≤ 8), medium (8–16), and severe (> 16). Sensitivity is reported as mean ± standard deviation across eight randomized test splits.

Reader ID.	Low	Medium	Severe
1	1.9 ± 1.7	15.7 ± 5.2	47 ± 8.3
2	58.4 ± 6.5	80.1 ± 8.2	92.2 ± 4.3
3	26.5 ± 4.7	68 ± 8.2	77 ± 10.4
4	7.3 ± 3.2	38.6 ± 7.7	61 ± 5.9
Readers Consensus	21.5 ± 5	59.5 ± 8.1	74.1 ± 6.9
DL	60.7 ± 13.5	74 ± 11.1	90.5 ± 8.3

In the low severity group, where radiographic signs are subtle, the model achieved a sensitivity of 60.7 ± 13.5%, substantially outperforming the consensus (21.5 ± 5%) and most individual readers (Reader 1: 1.9 ± 1.7%, Reader 3: 26.5 ± 4.7%, Reader 4: 7.3 ± 3.2%). Only Reader 2 showed comparable performance (58.4 ± 6.5%). For medium severity cases, the model maintained high sensitivity (74 ± 11.1%), exceeding the consensus (59.5 ± 8.1%) and three readers, again performing close to Reader 2 (80.1 ± 8.2%). In severe cases, the model reached 90.5 ± 8.3%, comparable to Reader 2 (92.2 ± 4.3%) and higher than both the consensus (74.1 ± 6.9%) and the other readers. Overall, the proposed DL system demonstrated strong and consistent performance across all severity levels, particularly outperforming human readers in low and medium cases. These results highlight its potential to support early detection and improve diagnostic reliability across varied disease presentations.

#### Benchmark selection and ablation study.

We compared several benchmark CNN models, including VGG19 [48], ResNet50 [49], InceptionV3 [50], Xception [51], and InceptionResNetV2 [42], using five-fold cross-validation with consistent data partitioning and preprocessing. All models were trained and evaluated on thorax regions extracted from CXRs. As shown in Table 10, VGG19 exhibited higher recall for non-COVID-19 viral pneumonia cases, and ResNet50 showed higher recall in identifying normal cases. Nonetheless, both models underperformed on other key metrics. InceptionResNetV2 consistently delivered better performance across all diagnostic categories, making it the selected backbone for our DL framework.

**Table 10:** Performance comparison across architectures for differentiating COVID-19 from other viral pneumonia and normal cases. The presented results are the Mean ± SD of the obtained results through a five-fold cross-validation process. The best results have been highlighted in bold. The results have been reported for the first diagnosis scenario.

Method	COVID-19			non-COVID-19 viral pneumonia			Normal			Accuracy (%)
	Recall (%)	PPV (%)	F1-score (%)	Recall (%)	PPV (%)	F1-score (%)	Recall (%)	PPV (%)	F1-score (%)	
VGG19 [48]	52.94 ± 5.5	63.47 ± 4.5	57.53 ± 3.7	62.48 ± 14.4	67.17 ± 6.5	63.46 ± 6.3	**83.06 ± 10.8**	71.32 ± 8.2	75.87 ± 3.1	66.78 ± 3.3
ResNet50 [49]	58.23 ± 12.8	71.39 ± 9.3	62.56 ± 4.9	**74.84 ± 11.8**	68.27 ± 4.5	70.71 ± 3.4	76.05 ± 4.5	75.38 ± 4.8	75.52 ± 1.9	70.45 ± 0.9
InceptionV3 [50]	58.4 ± 8.3	73.02 ± 6.4	64.35 ± 4.2	73.28 ± 6.4	67.29 ± 3	70.04 ± 3.5	79.87 ± 5	75.93 ± 7.4	77.5 ± 2.7	71.24 ± 2.6
Xception [51]	64.13 ± 6.3	69.25 ± 5.9	66.18 ± 1.9	70.55 ± 7.9	73.5 ± 3.1	71.67 ± 2.8	82.42 ± 5.7	76.14 ± 6.3	78.03 ± 2.2	72.77 ± 1.5
Inception-ResNetV2 [42]	**70.94 ± 8.1**	**71.68 ± 7.9**	**70.78 ± 4.5**	72.67 ± 6.3	**73.88 ± 3.7**	**73.08 ± 3.1**	79.11 ± 3.5	**77.43 ± 2.4**	**78.2 ± 1.6**	**74.04 ± 2.5**

We performed a series of experiments as detailed in Table 11 to evaluate the effectiveness of each component within the DL system. Table 12 shows the impact of these components in the first diagnostic scenario. Segmenting the thorax region significantly improved performance by directing the model’s focus to relevant areas, reducing false positives, and increasing computational efficiency by masking irrelevant regions. Additionally, the two-step classification and hierarchical transfer learning contributed to more accurate and balanced results. Table 13 demonstrates the impact of developmental steps on the performance of the DL diagnostic model in the second scenario, confirming that both the two-step classification and transfer learning consistently enhanced accuracy and F1-scores for all classes.

**Table 11:** Design of experiments. TL: Transfer Learning.

	Whole CXR	Thorax Region Segmentation	One-step classification	Two-step classification	Multi-step TL
Exp. I	✓		✓		
Exp. II		✓	✓		
Exp. III		✓		✓	
Exp. IV		✓		✓	✓

**Table 12:** Ablation study in the firs diagnosis scenario (excluding COVID-19 CXRs with no evidence of infection) over the five-fold cross-validation approach.

Exp. No.	COVID-19			non-COVID-19 viral pneumonia			Normal			Accuracy (%)
	Recall (%)	PPV (%)	F1-score (%)	Recall (%)	PPV (%)	F1-score (%)	Recall (%)	PPV (%)	F1-score (%)	
Exp. I	64.9 ± 11.9	68.12 ± 7.7	65.34 ± 2.4	70.1 ± 14.6	70.02 ± 3.6	69.21 ± 6.7	78.09 ± 7.1	77.73 ± 2.5	77.68 ± 2.5	71.33 ± 2.4
Exp. II	70.94 ± 8.1	71.68 ± 7.9	70.78 ± 4.5	72.67 ± 6.3	73.88 ± 3.7	73.08 ± 3.1	79.11 ± 3.5	77.43 ± 2.4	78.2 ± 1.6	74.04 ± 2.5
Exp. III	74.57 ± 5.7	69.52 ± 3.7	71.72 ± 1.1	70.67 ± 8	**76.02 ± 5.3**	72.79 ± 2.2	80.76 ± 2.8	80.02 ± 3.4	80.66 ± 1.1	75.26 ± 0.7
Exp. IV	**74.58 ± 3.4**	**73.77 ± 3.2**	**74.07 ± 1.5**	**73.29 ± 5.6**	75.48 ± 3.2	**74.23 ± 3**	**81.4 ± 3.2**	**80.11 ± 2.6**	**80.67 ± 0.9**	**76.44 ± 1.5**

**Table 13:** Ablation study in the second diagnosis scenario (including all 808 COVID-19 CXRs) over the five-fold cross-validation approach. In all experiments, the segmented thorax regions have been used as the model input.

Exp. No.	COVID-19			non-COVID-19 viral pneumonia			Normal			Accuracy (%)
	Recall (%)	PPV (%)	F1-score (%)	Recall (%)	PPV (%)	F1-score (%)	Recall (%)	PPV (%)	F1-score (%)	
Exp. II	58.2 ± 11.1	**71.1 ± 8.7**	63.1 ± 5.8	66.7 ± 7.6	**73.9 ± 4.5**	69.8 ± 4	83.8 ± 6.4	66.4 ± 2.9	74.0 ± 2.5	69.5 ± 2.8
Exp. III	59.6 ± 10.3	69.2 ± 4.7	63.4 ± 4.2	74.0 ± 7.6	71.5 ± 5.6	72.3 ± 1.8	**78.3 ± 7.5**	**73.1 ± 5**	75.3 ± 3	70.8 ± 0.6
Exp. IV	**62.2 ± 6.4**	70.8 ± 4.7	**66 ± 3.9**	**75.4 ± 7.5**	73.2 ± 5.3	**74 ± 4.2**	78.3 ± 8.8	73.1 ± 5.3	**75.3 ± 4.9**	**72.1 ± 2.5**

#### External validation of the diagnostic module.

To assess the generalizability of the diagnostic module, we conducted external validation on the COVIDGR-1.0 dataset [29], which contains 426 COVID-19 CXRs and 426 normal cases. This dataset includes only high-quality posteroanterior views, providing a reliable benchmark for binary classification models. For comparison, we included previously reported results for COVIDNet-CXR [35] and COVID-CAPS [36], which were also evaluated directly on this dataset. As presented in Table 14, our model achieved an overall accuracy of 73.59%, with balanced performance across classes: F1-scores of 75.94% (normal) and 70.74% (COVID-19). In contrast, COVIDNet-CXR and COVID-CAPS achieved lower accuracies of 49.76% and 47.66%, respectively.

**Table 14:** External validation results of the diagnostic model on the COVIDGR-1.0 dataset, compared with previously published models (COVIDNet-CXR and COVID-CAPS). Performance metrics include class-wise recall, precision, F1-score, and overall accuracy.

Model	Normal			COVID-19			Accuracy (%)
	Recall (%)	Precision (%)	F1-score (%)	Recall (%)	Precision (%)	F1-score (%)	
COVINet-CXR [35]	0.23	16.00	0.45	99.29	33.54	50.14	49.76
COVID-CAPS [36]	26.30	45.81	33.42	69.01	48.36	56.87	47.66
The proposed diagnostic module	83.33	69.74	75.94	63.85	79.30	70.74	73.59

We further validated the full diagnostic pipeline using a multi-source external dataset [52], randomly selecting 1,000 normal, 1,000 COVID-19, and all non-COVID-19 viral pneumonia cases (1345 cases). The model achieved 70.7% overall accuracy, with F1-scores of 70.7% (normal), 77.1% (non-COVID-19 viral pneumonia), and 63.4% (COVID-19). These results indicate that the model generalizes reasonably well to unseen data, likely due to its reliance on thorax-segmented regions, allowing it to focus on relevant anatomical features and reducing noise from surrounding non-informative areas.

### Performance of COVID-19 severity scoring module vs radiologists

The severity scoring model was trained using the consensus of all four readers as the ground truth. To evaluate the model’s sensitivity to inter-reader variability during testing, we established multiple ground truth configurations based on different subsets of three readers: (i) readers 1, 2, and 3; (ii) readers 1, 3, and 4; (iii) readers 1, 2, and 4; (iv) readers 2, 3, and 4; and (v) all four readers. For further analysis, test set images were stratified into three groups based on severity score (SI) ranges: SI ≤ 8, 8 < SI ≤ 16, and SI > 16, allowing us to assess model performance across different severity levels.

As shown in Table 15, the DL system achieved a Pearson correlation of 93% ± 0.7 and an MAE of 2.35 ± 0.14 across five-fold cross-validation when using the consensus of all four readers. When using different subsets of three readers as the ground truth, the Pearson correlation remained high (92.32%−92.76%), and MAE ranged from 2.38 to 2.54, demonstrating the model’s robustness to variations in reader consensus. Furthermore, the results showed MAE increased with higher severity scores, with values of approximately 1.7 for SI ≤ 8, 2.5 for 8 < SI ≤ 16, and 3.9 for SI > 16. Given that MAE interpretation depends on the data range, this pattern suggests that the model’s prediction error remains proportionate across severity levels, indicating consistent performance across different degrees of disease severity.

**Table 15:** Evaluation of the severity scoring model’s robustness to inter-observer variability. The model was trained using the average scores from four radiologists. During testing, one radiologist was excluded at a time, and performance was compared against the remaining consensus. Results include overall Pearson correlation and MAE, along with subgroup MAEs across three severity levels: Group 1 (SI ≤ 8), Group 2 (9 ≤ SI < 16), and Group 3 (SI ≥ 16). Pearson Co.: Pearson correlation. MAE: mean absolute error. SI: Severity score Index.

Consensus	Pearson Co. (%)	MAE	MAE		
			Group1 (SI <= 8)	Group2 (9 <= SI < 16)	Group3 (SI >= 16)
1,2,3	92.76 ± 0.67	2.54 ± 0.1	1.71 ± 1.4	2.75 ± 1.95	4.37 ± 3.13
1,3,4	92.52 ± 0.91	2.44 ± 0.14	1.85 ± 1.51	2.56 ± 1.94	4.11 ± 3.13
1,2,4	92.32 ± 0.72	2.38 ± 0.15	1.83 ± 1.59	2.85 ± 2.11	3.5 ± 2.72
2,3,4	92.32 ± 0.54	2.46 ± 0.12	1.76 ± 1.53	2.59 ± 2	4.27 ± 3.3
1,2,3,4	93 ± 0.68	2.35 ± 0.14	1.7 ± 1.42	2.53 ± 2	3.88 ± 2.98

**Fig 11:**
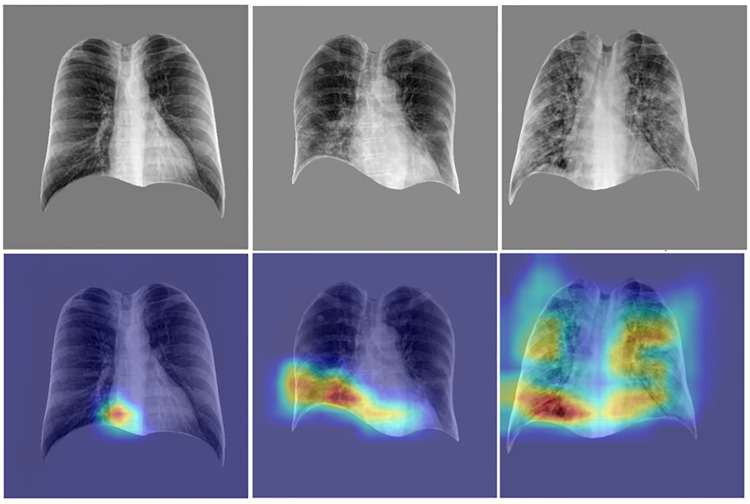
Representative examples of COVID-19 CXRs at varying severity levels alongside corresponding model attention maps. The first row shows thoracic regions extracted from the CXRs, while the second row presents Grad-CAM heatmaps highlighting areas that most influenced the model’s severity predictions. From left to right: a low severity case (SI ≤ 8; GT = 0; MP = 2.4), a medium severity case (8 < SI ≤ 16; GT = 9; MP = 9.5), and a high severity case (SI > 16; GT = 31; MP = 28.1). The heatmaps demonstrate an increasing focus on widespread pathological regions with rising severity, indicating that the model’s attention aligns with clinical patterns of disease progression. (SI: severity index; GT: ground-truth severity score; MP: model prediction severity score).

To further assess the model’s behavior across severity levels, Fig 11 presents representative COVID-19 CXRs and their corresponding Grad-CAM heatmaps. In low severity cases, the heatmaps show minimal activation, whereas medium and high severity cases demonstrate progressively stronger and more extensive attention patterns, particularly over regions of pulmonary opacities. These results indicate that the model’s predictions are guided by clinically relevant features associated with COVID-19 disease progression.

**Fig 12:**
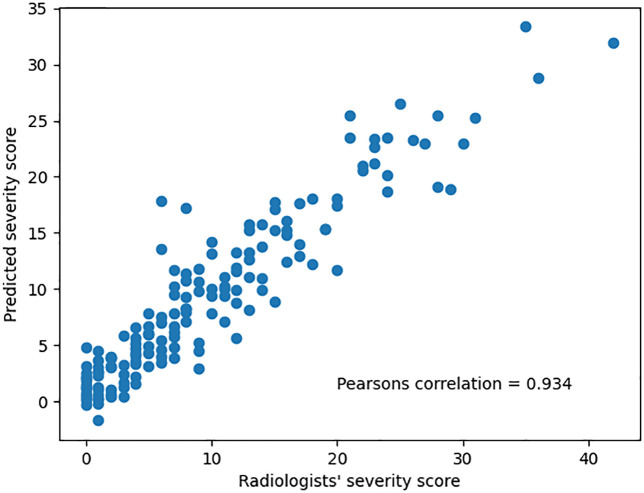
DL-Predicted severity scores versus consensus radiologists’ severity scores. Consensus radiologists’ severity scores are the average of severity scores evaluated by four radiologists.

The inter-rater reliability among radiologists, measured by ICC (2, k), was 0.804 (95% CI: 0.68–0.87), indicating good agreement. Although radiologists showed consistent severity scoring for COVID-19 CXRs, some variability remained, highlighting the potential role of the DL model in supporting more standardized assessments. Fig 12 shows a scatter plot comparing the DL-predicted severity scores to the radiologists’ consensus on the test set, demonstrating a strong correlation between the two.

#### External validation of the DL severity scoring module.

**Fig 13:**
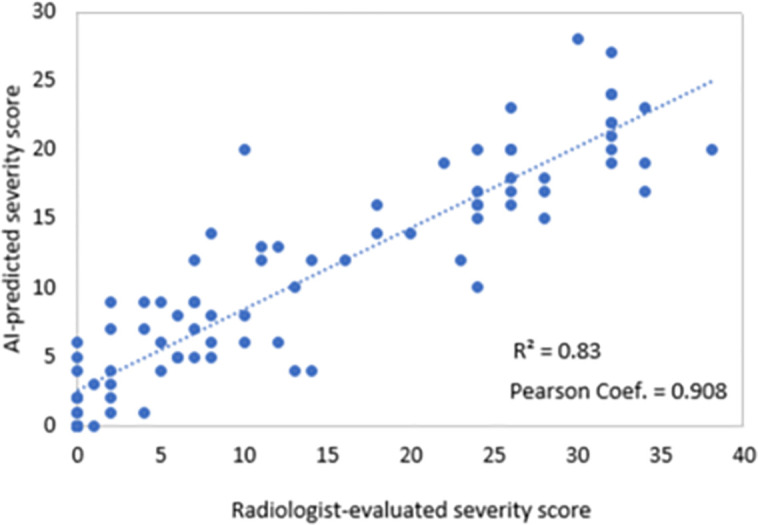
External validation of DL severity scoring module on 86 COVID-19 CXRs from a publicly available dataset.

To assess our model’s generalizability on unseen data, we selected a subset of 90 COVID-19 CXRs from a publicly available dataset [35]. First, the thorax segmentation module was applied to delineate the thorax masks. A non-specialist’s preliminary evaluation of the segmented thorax masks confirmed acceptable segmentation in 86 CXRs. The thorax regions were extracted by element-wise multiplication of the CXRs and masks and then fed into the severity scoring module. Predicted scores below zero, due to the model’s linear output activation, were adjusted to zero in a post-processing step. All 86 external CXRs were evaluated and scored by one of our radiologists (reader 2). Fig 13 illustrates the scattered plot of DL-predicted severity scores against the radiologist’s evaluations. The results revealed a Pearson correlation of 90.8% and an MAE of 4.95 ± 4.4, indicating a high level of agreement between the DL predictions and the radiologist’s assessments.

## Discussion

In this study, we developed a DL framework to differentiate COVID-19 from non-COVID-19 viral pneumonias and normal cases using baseline CXRs, and to quantify disease severity. We also compared the DL system’s performance with a panel of four radiologists in both diagnostic classification and severity scoring tasks.

Distinguishing COVID-19 from other viral pneumonias using CXRs is particularly challenging due to overlapping radiographic features. Typical radiographic features of COVID-19 include peripheral ground-glass opacities, predominantly in the mid and lower lung zones, along with coarse horizontal linear opacities and, less frequently, consolidations [39]. However, atypical appearances, such as patchy, bronchocentric, or diffuse ground-glass opacities and reticulonodular infiltrates, are often indistinguishable from those of non-COVID-19 viral pneumonias. Additionally, a significant portion of baseline CXRs may appear normal or show only subtle findings [10,44], with radiographic findings typically peaking around 10 days after symptom onset [53]. These diagnostic complexities were reflected in the moderate inter-rater agreement observed in our study (Fleiss’ Kappa = 0.52), reinforcing the potential value of a DL system for offering standardized assessments and reducing variability in radiologic interpretation.

To better assess the diagnostic performance of both the DL model and radiologists, we defined two scenarios: one excluding COVID-19 CXRs without evidence of infection and another including all COVID-19 CXRs. The DL model outperformed the radiologists’ consensus, achieving accuracies of 76.4% and 72.1%, with COVID-19 F1 scores of 74% and 66% in the first and second scenarios, respectively. In comparison, the consensus yielded lower accuracies of 71.8% and 68.2% and COVID-19 F1 scores of 61.3% and 54.5%. Individual radiologist performance varied significantly, with accuracies ranging from 59.1%−78.6% and 56.3%−74.9%, and COVID-19 F1 scores from 30.4%−78.9% and 26.4%−72.4% in the two scenarios. These variations underscore the complexity of the diagnostic task, while the DL model’s consistent performance suggests its potential to support early diagnosis.

Focusing on baseline CXRs to diagnose COVID-19 from other viral pneumonia distinguishes our work from existing research. While some studies explored differentiation from other viral pneumonias [21,22], they often lacked timing information or relied on mixed-stage imaging. Other research has focused on CT-based comparisons [54–56], or on differentiating COVID-19 from CAP [57,58], COVID-negative cases [59,60], and CAP and normal CXRs [35,61], often utilizing later-stage imaging.

Our DL model achieved an AUC of 89% for differentiating COVID-19 from non-COVID-19 viral pneumonia in the first diagnostic scenario. In comparison, Wang et al. [11] reported an AUC of 96.6% for distinguishing COVID-19 CXRs from CAP, which dropped to 86.7% against other viral pneumonias and to 81.7% for non-severe COVID-19 cases. In their study, severity was defined based on physiological criteria such as oxygen saturation. While these results highlight the complexity of distinguishing COVID-19 CXRs from other viral pneumonias, particularly non-severe cases, the use of non-imaging-based severity criteria and the lack of CXR timing limit direct comparison with our approach. Table 16 summarizes the AUC values reported in previous studies and our proposed model across various pneumonia detection tasks, where all studies involved close collaboration with radiologists for data interpretation and validation.

**Table 16:** Summary of reported AUC values from previous studies and the proposed model across different CXR-based pneumonia detection tasks. All studies had close collaboration with radiologists for data interpretation and validation.

Ref.	Classification task	Dataset size	Reported AUC	Evaluation strategy
[11]	COVID-19 vs Pneumonia (Bacterial + Viral)	164 COVID-19, 630 Pneumonia	0.966	Hold test set
[11]	COVID-19 vs Other Viral Pneumonia	164 COVID-19, 190 Other Viral Pneumonia	0.867	Hold test set
[11]	Severe COVID-19 vs Other Viral Pneumonia	Unspecified	0.913	Hold test set
[11]	Non-severe COVID-19 vs Other Viral Pneumonia	Unspecified	0.817	Hold test set
[17]	Normal vs Pneumonia (COVID-19 + Bacterial)	60 Normal + 120 Pneumonia	0.913	External test set
[20]	Normal vs COVID-19	105 Normal + 67 COVID-19	0.714	External test set
Proposed Model	Normal vs Pneumonia (COVID-19 + Other Viral)	803 Normal, 1744 Pneumonia	0.93	Hold test set
Proposed Model	COVID-19 vs Other Viral Pneumonia	808 COVID-19, 936 Other Viral Pneumonia	0.89	Hold test set

For severity scoring, our model showed robust agreement with expert annotations, achieving Pearson correlations of 92.3%–93% and mean absolute errors (MAE) of 2.35–2.54. In comparison, previous studies [11] reported lower correlation (e.g., 0.81) and higher MAE (e.g., 8.64) using broader or different scoring scales. Table 17 presents a summary of severity scoring performance across studies. Our model’s scores reflect not only the extent of lung involvement but also the radiographic patterns, often associated with the disease stage, offering a nuanced clinical assessment. Moreover, external validation on 86 public COVID-19 CXRs yielded a Pearson correlation of 90.8%, indicating good generalizability despite variations in imaging conditions. While our model does not explicitly incorporate temporal information, the predicted severity scores inherently reflect the extent of lung involvement and radiographic patterns commonly associated with disease progression. Although longitudinal analysis is beyond the scope of this study, the model’s consistent performance across a broad range of severity levels, along with its ability to provide clinically meaningful severity estimates, suggests that the proposed framework could serve as a foundation for future research on tracking disease progression over time. Reliably assessing the severity of COVID-19 pneumonia on a baseline CXR at the early stages of the disease may have predictive implications for the clinical outcomes of these patients, which is an area of research that we will explore further.

**Table 17:** Comparison of severity scoring performance across prior studies and the proposed severity scoring module. MAE: Mean Absolute Error.

Study/ Model	Scoring Range	Pearson Correlation	MAE	Evaluation strategy
[11]	0–48	0.81	8.64	External validation
[14]	0–18	0.86	1.73	Hold test set
[31]	0-8 Geographical extents 0-8 Lung Opacity	0.86–0.861	Not reported	Hold test set
Proposed DL Model	0–48	0.923–0.930	2.35–2.54	Hold test set
Proposed DL Model (external validation)	0–48	0.908	4.95	External validation

While our results are promising, several limitations should be considered. First, the model was trained using PCR results as ground truth labels for pneumonia diagnosis, which may have limited sensitivity due to sampling error. Additionally, there are limitations inherent to the retrospective nature of the study. While the dataset includes patient age and sex, the absence of racial and ethnic annotations limits conducting a comprehensive bias analysis. However, the data were collected at a hospital in Toronto, a city with a diverse and multicultural population, suggesting inherent demographic variety. Although the number of CXRs is relatively balanced across diagnostic categories, we employed bootstrapped resampling during evaluation to assess model performance under varying class distributions. Additionally, subgroup analyses by age, sex, and severity confirmed consistent model performance across these categories.

Despite being trained on data from a single institution, our diagnostic model showed reasonable performance on external datasets, suggesting generalizability. However, the lack of multi-center training data remains a limitation. While several public CXR datasets are available, they often lack balanced representation of all diagnostic categories from the same clinical source, which can lead models to distinguish classes based on center-specific artifacts. Even when domain adaptation techniques [62] are applied, imbalanced or incomplete datasets may cause the model to rely on non-clinical cues. Future work will focus on incorporating well-balanced, multi-class datasets from multiple institutions and applying domain adaptation techniques to enhance robustness across varied clinical environments.

The clinical impact of misclassification is an important consideration when integrating AI tools into diagnostic workflows. For instance, misidentifying COVID-19 cases for non-COVID viral pneumonia may delay isolation or appropriate treatment, increasing the risk of transmission. Conversely, misclassifying non-COVID cases as COVID-19 could lead to unnecessary isolation and resource use. Labeling normal CXRs as pneumonia may trigger avoidable diagnostic procedures, while missed pneumonia cases could delay treatment and worsen outcomes. These scenarios highlight the importance of thoughtful AI integration into clinical settings.

Safe and effective AI integration into healthcare must prioritize patient safety, transparency, and accountability. Our model is designed as a clinical decision-support tool, not a replacement for expert judgment. As responsibility for diagnostic outcomes continues to rest with human practitioners, AI tools must offer interpretable and reliable outputs to aid informed judgment. Before deployment, such systems require rigorous validation in real-world settings and must meet regulatory standards, including those set by the U.S. Food and Drug Administration (FDA), which emphasize explainability and clinical utility [63]. Establishing clear boundaries for AI recommendations and documentation practices will be essential as regulatory and medico-legal frameworks evolve.

To assess the model’s clinical utility, a prospective observational study could be conducted where the model is integrated into radiology workflows as a decision support tool. Radiologists would review chest X-rays both with and without AI assistance, allowing for comparison of diagnostic performance, inter-reader agreement, and reporting time. Key outcome measures could include diagnostic accuracy, sensitivity for early-stage COVID-19, consistency in severity scoring, and the influence of AI assistance on clinical decision-making. Additionally, the model’s impact could be evaluated in different clinical contexts, such as emergency departments or resource-limited settings, to examine its usefulness in triage or rapid diagnosis. Collecting feedback from clinicians on usability and interpretability (e.g., via visual explanations or confidence scores) would also provide valuable insight into its adoption potential. These steps would help ensure the model’s robustness and relevance in practical healthcare scenarios.

Automated detection of suboptimal CXRs using AI helps identify technical issues such as improper exposure, patient mispositioning, or imaging artifacts that may affect diagnostic accuracy. Incorporating such quality control steps ensures that only diagnostically reliable images proceed to analysis, enhancing both model performance and workflow efficiency [64]. In our study, low-quality images due to technical errors or positioning issues were visually reviewed and excluded. We also employed real-time data augmentation, helping the model generalize to minor variations and imperfections. For deployment, the thorax segmentation module serves as a built-in quality check. With human-in-the-loop oversight, failure to accurately segment the thoracic region, often indicative of suboptimal image quality, results in exclusion from downstream analysis. Looking ahead, integrating AI-based suboptimal image detection tools may further enhance the reliability of the DL pipelines.

In summary, our DL framework demonstrates strong potential as a decision-support tool for early COVID-19 diagnosis and severity scoring using baseline CXRs. It shows robust performance across subgroups, aligns well with expert evaluations, and generalizes reasonably to external data, positioning it as a valuable tool for assisting radiologists in real-world settings.
